# Intraoral Scan Accuracy and Time Efficiency in Implant-Supported Fixed Partial Dentures: A Systematic Review

**DOI:** 10.7759/cureus.48027

**Published:** 2023-10-31

**Authors:** Fawaz Pullishery, Wayel Huraib, Abdullah S Alruhaymi, Wabel Abdulrahman Alharandah, Elaf Waleed AlDara, Muath Mohammed Benten, Dina Mansour Alassaf, Wafa Mohammed A Babatin, Nagam Mohammed M Mohsen

**Affiliations:** 1 Community Dentistry and Research, Batterjee Medical College, Jeddah, SAU; 2 Fixed Prosthodontics, Dentistry Program, Batterjee Medical College, Jeddah, SAU; 3 General Dentistry, Ministry of Health, North Obhur, Jeddah, SAU; 4 General Dentistry, Ministry of Health, Almurjan, Jeddah, SAU; 5 General Dentistry, King Abdulaziz University, Jeddah, SAU; 6 General Dentistry, Ministry of Health, Riyadh, Riyadh, SAU; 7 General Dentistry, Dentistry Program, Batterjee Medical College, Jeddah, SAU; 8 General Dentistry, Self Experts Center, Jeddah, SAU

**Keywords:** implant-supported fixed prosthesis, dental implantology, time efficiency, scan accuracy, intraoral scanner

## Abstract

The digital implant impression technique (DIT) and conventional implant impression technique (CIT) workflows in implant-supported fixed partial dentures (FPDs) have not been extensively compared in prior studies. Moreover, there is no agreement on the more accurate method that entails less time in the laboratory and during the clinical phases of fabrication and delivery of the prosthesis, respectively. This review aimed to assess the precision of the imaging procedure and overall fabrication time of the DIT and CIT for the implant-supported FPDs. An electronic search was performed using PubMed, Scopus, EMBASE, Cochrane Oral Health Group, and Dentistry and Oral Science Source databases through EBSCO for relevant studies from January 2014 to April 2023. Following the preliminary screening, the studies that met the inclusion criteria underwent full-text review in accordance with the Preferred Reporting Items for Systematic Reviews and Meta-Analyses guidelines. The Cochrane Collaboration risk of bias appraisal tool and Newcastle-Ottawa scale were applied to assess the quality of randomized controlled trials (RCTs) and non-randomized prospective clinical studies, respectively. The initial search yielded 332 studies, and after excluding duplicates, 241 papers were available for screening. Titles and abstracts were reviewed, and 97 articles were chosen for full-text review by two authors independently. Furthermore, 89 articles were excluded in compliance with the PICOS question, and eight studies were chosen for qualitative analysis. Hence, the review comprised two RCTs and six prospective clinical studies. The time efficiency of the implant-supported FPDs was examined in four investigations, three of which used the Trios 3 scanner and one used the Intero scanner. The three-dimensional accuracy of DIT and CIT was compared in six clinical comparative studies. One of the RCTs was rated to have a high risk of bias and the other with a moderate quality of evidence. The six prospective studies were rated to have high-quality of evidence. The findings of this review indicate the prospective applicability of future intraoral scanning systems. The DIT was reported to be outstanding in terms of patient preferences and total fabrication time efficiency. Additional in vivo studies are needed to establish the therapeutic usefulness and time efficiency of integrating DIT in more comprehensive settings.

## Introduction and background

The degree of trueness and precision of implant impressions is especially important in prosthetic rehabilitation, which usually entails capturing the bodily structure and the precise positioning of endosseous implants, and the morphology of the peri-implant tissues. This, in turn, influences the degree of accuracy of the final prostheses [1,2]. Mechanical and biological ramifications result from a surface mismatch of the implant prosthesis due to an erroneous impression [3,4]. Several impression methods have been tested to accomplish a passive fit with the implant framework and implant body. Diverse impression techniques involving the transfer technique or pick-up method, dual-arch impression technique, and coping modification were established to improve the accuracy of impression techniques [3]. Despite being frequently used in clinical settings for several decades, the conventional implant impression method (CIT) has a few limitations, notably the need for material preparation, impression distortion, sensitive technique, time prerequisite, unfavorable taste, and discomfort for the patient [2,5].

The digital implant impression technique (DIT) has resolved most of the limitations of the CIT by removing prerequisites for impression materials, enhancing patient comfort, curbing the amount of time needed, and providing the capability to retain digital data [6,7]. When contrasted with full-arch scanning, quadrant scans are notably more efficient in terms of time and are less susceptible to accuracy variations. According to prior studies, accuracy decreases as the extent of the scanned region increases. A higher risk of accruing flaws is observed in intraoral scanning (IOS) of completely edentulous subjects owing to an array of parameters, including salivary secretion, reflective restorations, mobile mucosa, surface traits, and various scanning methods [2,6].

Complete DIT includes the real-time three-dimensional (3D) capture of the unique patient scenario within the oral cavity using IOS, computer-aided design (CAD) using dental software systems for swift prototyping such as milling or 3D printing (computer-aided manufacturing (CAM)), and professional delivery of dental prostheses. The construction, transfer, and subsequent analysis of the obtained IOS information into a Standard Tessellation Language (STL) file are essential measures in DIT. The digital approach is generally related to the fabrication of physically robust and precise manufacture of the monolithic zirconia prosthesis in an expedited process with a decreased requirement for physical human contact [8,9]. Currently, there is a scarcity of intraoral implant impressions that enable a flawless 3D transfer to a reference framework. In prosthodontic rehabilitation, these errors are corrected by intraoral bonding of a tertiary framework. The acceptable range of accuracy for implant impressions varies. While the early literature reported a gap of 10 μm or less between the framework and abutment, a gap of no more than 150 μm has also been considered appropriate. This issue is not exclusive to DIT and is also widely recognized to exist in CIT [10].

The impression accuracy of DIT in vivo has not been extensively studied yet [1,11]. Further, the DIT and CIT workflows in implant-supported fixed partial dentures (FPDs) have not been comprehensively compared in prior studies, and, as a result, there is no agreement on which method is more accurate and entails less time in the laboratory and during clinical phases of fabrication and delivery of the prosthesis. This review aimed to determine the accuracy of the imaging and overall fabrication time of the DIT and CIT for implant-supported FPDs.

## Review

Methodology

The review was carried out in accordance with the Preferred Reporting Items for Systematic Reviews and Meta-Analyses (PRISMA) standards [12]. A structured question was developed based on the PICOS criteria (patient, intervention, comparison, outcome, and study design).

The study population included partially edentulous patients with an implant-supported FPD. Studies involving DIT/IOS methods were included as the intervention. Studies discussing the CIT were included as the comparison. Study outcomes included measurement of accuracy, precision, or trueness of impression and total fabrication time taken to make the impression. Randomized controlled trials (RCT) or prospective clinical studies were considered for inclusion.

Focused Question

Is IOS a more accurate and less time-consuming method for fabricating the final prosthesis than CIT employed during implant-supported FDP rehabilitation?

Search Strategy

An electronic search was conducted using PubMed, Scopus, EMBASE, Cochrane Oral Health Group, and Dentistry and Oral Science Source databases through EBSCO for relevant studies published in the English language from January 2014 to April 2023. The following Medical Subject Headings phrases were applied: (“Implant Supported Fixed Denture Prosthesis” OR “Fixed Partial Denture” OR “Implant Prosthesis” OR “Implant Reconstruction” OR “Fixed Reconstruction” OR “Partially Edentulous” OR “Fixed Bridge” AND (“Intraoral Scan” OR “Digital Implant Impression Technique” OR “Digital Workflow”) AND (“Conventional Implant Impression Technique” OR “Conventional Workflow” OR “Analog Impression Technique”) AND (“Three-Dimensional Accuracy” OR “Transfer Accuracy” OR “Scan Accuracy” OR “Surface Misfit” OR “Precision” OR “Trueness” OR “Fabrication Time” OR “Time Efficiency”). A manual search of journals for relevant publications was also conducted. Google Scholar was used to search for any studies that were not selected in the aforementioned databases.

Selection Criteria for Eligible Studies

The following were the inclusion criteria to be fulfilled for studies to be considered eligible for inclusion in this systematic review: RCT or non-randomized prospective clinical studies (in vivo) encompassing patients receiving implant-supported FDPs who were partially edentulous; studies evaluating the workflows of DIT and CIT strategies, accuracy, precision, or trueness of the IOS; and/or studies evaluating the speed of DIT and DIT of FPDs. Studies that were published before 2014 were eliminated, including in-vitro studies, expert comments, review articles, technical reports, studies on animal models, and case reports/series.

Study Selection

Two reviewers independently reviewed each title and abstract during the initial evaluation. Following the preliminary screening, studies that met the inclusion criteria underwent full-text analysis. Additionally, the lists of references of the selected studies were explored for any relevant published articles. If there were any discrepancies between the reviewers, a third reviewer was consulted to validate the review process.

Quality Assessment

Applying the Cochrane Collaboration assessment tool for evaluating the risk of bias (RoB) for RCTs, two independent investigators executed a quality evaluation [13]. The tool addresses the seven distinct domains, encompassing concerns with sequence generation, allocation concealment, blinding of the participants and investigator, blinding of outcome evaluation, insufficient outcome measures, selective result reporting, and any other concerns. When all the criteria were satisfied, low RoB was assigned. When one of these domains was not fulfilled, medium RoB was allocated. When two or more domains were not met, a high RoB was designated.

The Newcastle-Ottawa scale (NOS) was utilized to appraise the quality of the reviewed non-randomized prospective clinical studies [14]. The NOS rates the selection, comparability, and study outcome domains with a maximum of four, two, and three stars, respectively. Studies were deemed to have a high methodological quality if they received an NOS score of at least six (maximum score = 9). NOS scores under four were deemed to carry a substantially high RoB. The divergences of opinion between the investigators were resolved through discussion.

Data Extraction

Two reviewers retrieved data independently from the included studies based on the predefined outcomes of interest for further qualitative analysis. The author names, publication year, the study design, sample size, demographic traits such as age and gender, the number of impressions or implants used, the purpose of the study, the type of IOS employed for scanning, the CIT material utilized, the number of implants used, the workflows of CIT and DIT, and the outcome assessment were extracted from the included studies. The review focused on the time required for the overall fabrication and completion of the CIT and DIT workflows, as well as the outcome measures of scan accuracy/precision/trueness in terms of measurement of variances in distance deviations or implant angulation. As previously stated, disagreements were resolved through discussion. In cases where additional details about the study methodology or any specific data were needed, the corresponding authors of the studies were communicated with.

Results

Study Selection

**Figure 1:**
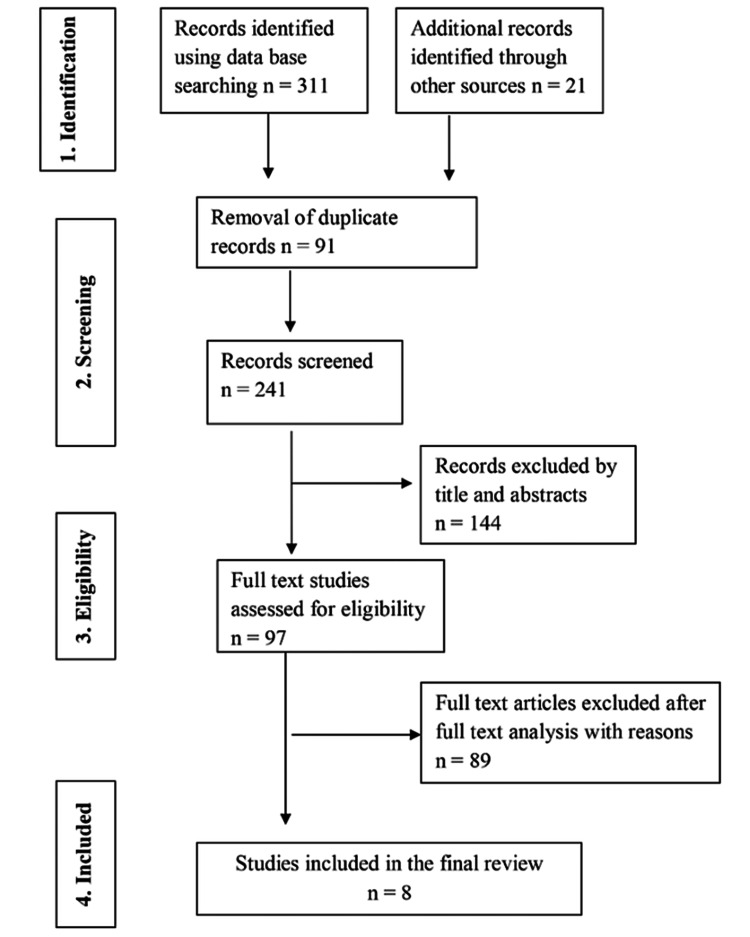
Preferred Reporting Items for Systematic Reviews and Meta-Analyses (PRISMA) flowchart of the included studies (adapted from PRISMA 2009 flow diagram).

The preliminary electronic search resulted in 311 articles, as shown in the PRISMA flow diagram (Figure 1).

The manual search resulted in the selection of 21 relevant articles from a total of 332 articles for the process of the review. After removing the duplicate records, 241 publications were subjected to the initial screening. The abstracts of 144 articles were discarded after a review of the titles and abstracts. Further, 97 articles were selected for full-text review by two investigators independently. Finally, eight studies were chosen for qualitative review [15-22]. Out of the eight studies, two were RCTs [16,17] and six were prospective clinical studies [15,18-22]. Kappa score for interexaminer reliability was 0.84 for title/abstract screening and 0.90 for the full-text review. Any disparities between the investigators were settled through consultation with a third reviewer.

Characteristics of Included Studies

Six prospective clinical trials and two RCTs were included in the review. The time efficiency of the implant-supported FPDs was examined in four investigations, three of which used the Trios 3 IOS [15,17] and one used the Intero IOS [22]. The 3D accuracy of DIT and CIT was compared in six clinical comparison studies [15,17-21]. In two trials, the effectiveness of the two approaches in terms of time, cost, and patient preferences were specifically contrasted [16,22]. Polyether [18,22] and polyvinylsiloxane [15-17,19-21] were utilized for CIT. Except for a study by Wismeijer et al., every study that was included used Trios 3 IOS [22]. Gintaute et al. evaluated the laboratory cross-mounting procedure employing implant-supported FPDs with 3D-printed implant frameworks for the complete digital workflow of two groups and gypsum implant master cast for the control group [17]. The clinical performance indicators comprising the clinical fit of the implant-supported FPDs requiring chairside adjustment time and the subsequent density variations of the prosthesis were also evaluated. The summary of the reviewed studies is presented in Table 1.

**Table 1:** A summary of the reviewed studies. CIT = conventional implant impression technique; DIT = digital implant impression technique; ZrO_2_ = monolithic zirconium oxide; FPD = fixed partial dentures; STL = Standard Tessellation Language

Author, year	Study design	Sample size	Number of implants/Impressions	Scanner system	Conventional impression material	Outcome measures
Hashemi et al., 2022 [15]	Prospective controlled crossover trial	10 patients with three-unit implant-supported FPDs in the posterior mandible	20 prostheses (10 prostheses in CIT and 10 prostheses in DIT)	Trios 3; 3Shape, Denmark	Polyvinylsiloxane-light body addition silicone (Panasil, Kettenbach GmbH & Co., Germany)	The accuracy and framework adaptation were comparable between the CIT and DIT. The fabrication time was substantially reduced using the DIT (178.2 ± 2.60 minutes) than the CIT (498.9 ± 4.50 minutes) workflow
Joda et al., 2021 [16]	Triple-arm crossover RCT	20 subjects with two Straumann tissue-level implant-supported FPDs in the maxillary or mandibular posterior region	60 monolithic ZrO_2_ FPDs by group 1 (IOS Trios 3 + Dental System Lab-Software), group 2 (IOS Virtuo Vivo + DWOS Lab-Software), and Control (CI, Gypsum Cast, Lab-Scan + Exocad Lab-Software)	Trios 3/3Shape (Group 1) and Virtuo Vivo/Straumann (Group 2)	The mixed analog-digital procedure with polyether impression and digitized gypsum cast (Impregum/3M ESPE)	Mean total work time was 97.5 ± 23.6 minutes for group 1, 193.1 ± 25.2 minutes for group 2, and 172.6 ± 27.4 minutes for controls with statistically significant differences
Gintaute et al., 2021 [17]	Triple-arm crossover RCT	20 patients (mean age of 63 years) with a three-unit FPD with two tissue-level implant systems (RN/WN, Institut Straumann AG, Basel, Switzerland)	60 monolithic ZrO_2_ FPDs by Group-1 (IOS Trios 3 + Dental System Lab-Software), group 2 (IOS Virtuo Vivo + DWOS Lab-Software), and control (CI, Gypsum Cast, Lab-Scan + Exocad Lab-Software)	Trios 3/3Shape (Group 1) and Virtuo Vivo/Straumann (Group 2)	The mixed analog-digital process with polyether impression and digitized gypsum cast (Impregum/3M ESPE)	The highest transfer success rate of laboratory cross-mounting was found in the original pairing of the implant-supported FPD model control (75%) and the lowest was in the implant-supported FPD control model group 2 (10%). Overall, 60% of implant-supported FPDs with group 1 workflow did not necessitate chairside adjustment compared to 50% for group 2 and 30% for the control group. The average of the sum of interproximal, pontic, and occlusal correction time was 2.59 ± 2.51 minutes (controls), 2.88 ± 2.86 minutes (group 1), and 3.87 ± 3.02 minutes (group 2)
Schmidt et al., 2021 [18]	Prospective clinical study	20 subjects with a minimum of three implants in either of the jaws in two different quadrants	39 cases were included (22 edentulous, 8 partially edentulous in the maxillary arch, the mean distance between implants 30.15 ± 11.18 mm; 6 edentulous, 3 partially edentulous in the mandibular arch, the mean distance between implants 33.19 ± 14.85 mm	Trios 3 pod	Polyether (Impregum Penta)	The point deviation for trueness and precision was expressed as mean ± SD (ISO 5,725). Insignificant differences in transfer accuracy were found between the CIT (0.045 ± 0.035 mm) and DIT (0.040 ± 0.029 mm) in the partially edentulous maxillary arch. A statistically significant difference between the CIT (0.046 ± 0.027 mm) and DIT (0.079 ± 0.050 mm) was observed in the mandibular arch
Rutkunas et al., 2020 [19]	Prospective clinical study	20 patients with two implant-supported FPDs	10 two-unit, 11 three-unit, and 6 four-unit FPDs supported by two implants (AnyOne, Megagen, Korea) were evaluated. Among these, three prostheses were in the anterior and 24 in the posterior region with the mean distance between implants of 14.3 ± 7 mm	Trios IOS (3Shape, Copenhagen)	Polyvinylsiloxane (Express, 3M ESPE, Minnesota, USA)	The inter-implant distance, angulation, and surface mismatch of scan body parameters were statistically significant (p < 0.05). Surface mismatch values for the Trios and D800 were 31.8 ± 25.6 µm (DIT) and 14.27 ± 21.5 µm (CIT) for the mesial implant, and 30.68 ± 28.7 µm (DIT) and 14.65 ± 19.7 µm (CIT) for distal implant scan bodies
Gedrimiene et al., 2019 [20]	Prospective clinical study	Six subjects with two implant-supported FPDs (Megagen, Daegu, Korea)	Seven two-unit, 11 three-unit, and six four-unit zirconia prostheses were fabricated with the mean inter-implant distance of 15.82 ± 5.66 mm	Trios 3 IOS (3Shape)	Polyvinylsiloxane (Express, 3M, Maplewood, MN, USA)	The mean differences in inter-implant distance (70.8 ± 59 µm), rotation (-2.0 ± 1.37), vertical shift (- 82.2 ± 61.7 µm), and surface mismatch (for DIT and CIT were 34.14 ± 36.69 µm and 14.19 ± 3.22 µm for the mesial implant and 34.24 ± 14.64 µm and 14.19 ± 2.29 µm for the distal implant scan bodies) differences were found to be statistically significant between CIT and DIT
Alsharbaty et al., 2019 [21]	Prospective clinical study	36 subjects	36 patients with two implant-supported FPDs (Implantium) in diameter between 3.8 and 4.8 mm and <20° difference in angulation between the two implants in posterior regions	Trios 3Shape IOS; Copenhagen	Polyvinylsiloxane-Soft putty and light-body (Panasil, Kettenbach GmbH & Co., Germany)	Measurement of distance deviation, angular and linear displacement (ΔD = 0.09 ± 0.02 mm, Δθ = 2.01 ± 0.33° and Δr = 0.16 ± 0.025 mm) revealed that the pick-up procedure produced lesser mean deviation values than the transfer technique and the DIT (p < 0.001)
Wismeijer et al., 2014 [22]	Prospective clinical study	30 subjects	41 implants (Straumann tissue level) in the posterior region	iTero IOS	Polyether (Impregum; 3M Espe, MI, USA)	The overall time taken for the CIT technique was found to be lesser than that of the DIT (p < 0.05)

The objectives and detailed workflow of each study are presented in Table 2.

**Table 2:** Workflow and objectives of the reviewed studies. CIT = conventional implant impression technique; DIT = digital implant impression technique; ZrO_2_ = monolithic zirconium oxide; FPD = fixed partial dentures; STL = Standard Tessellation Language; CMM = coordinate measuring machine

Author, year	Study objective	Workflow
Hashemi et al., 2022 [15]	To contrast the clinical outcomes of DIT and CIT workflows for the production of three-unit implant-supported FPDs in regards to the precision of the impression, fitness, and amount of time required	The CIT and DIT were made from polyvinylsiloxane and IOS, respectively, and the frameworks were produced from cobalt-chromium and zirconia. The standard master casts and reference models were captured using a 2 µm accuracy scanner. The CIT and DIT STL files were superimposed on the default model file. To assess the accuracy of the CIT and DIT, linear and angular alteration was measured. The amount of time needed to accomplish both kinds of workflows was documented
Joda et al., 2021 [16]	To examine the time efficiency along with manufacturing costs of three distinct workflows for three-unit monolithic ZrO_2_ implant-supported FPD	All FPDs were manufactured using CAD-CAM and were made of monolithic ZrO_2_ (VITA Super Translucent Multicolor, Germany) bonded to pre-fabricated titanium abutments (Variobase, Basel). Employing the envelope method the sequence of impression-taking for the three groups and the prosthetic try-in was assigned at random. The key result was time efficiency, which was characterized as the overall duration in minutes for clinical care and laboratory performance
Gintaute et al., 2021 [17]	To investigate the laboratory and clinical utility of monolithic ZrO_2_ implant-supported FPDs with two complete DIT workflows and one mixed analog-digital procedure	The study included implant impressions for clinical registration, CAD/CAM manufacture of implant-supported FPD, and try-in/delivery. The practicality of laboratory cross-mounting of each FPD was evaluated, as well as clinical fit and adjustment duration concerning interproximal surfaces, pontic areas, and occlusal regions. All 60 prostheses were tested onto the conventionally created gypsum implant castings (control group) and over the 3D-printed implant model (groups 1 and 2) using the three workflows, which yielded nine viable pairings for laboratory cross-mounting
Schmidt et al., 2021 [18]	To contrast the rate of the transfer accuracy of CIT and DIT using a novel reference key method	Individualized reference keys were produced and reversibly implanted. There were 86 implants employed in total: 18 ProActive Straight (Neoss, Germany), 48 Narrow Crossfit, and 20 Regular Crossfit Bone Level (Straumann, Germany). CIT and DIT were used to create the impressions. To compare the point of deviations of the trueness of the typical and digital models, the center points of the implant of both techniques were calculated utilizing coordinate-measuring equipment or three-dimensional evaluation software and superimposed with the reference key locations
Rutkunas et al., 2020 [19]	To contrast the clinical accuracy of CIT and DIT	A D800 laboratory scanner was used to scan the master casts. STL files were exported from CIT and DIT procedures for comparison. The distance between the center points, angulation, rotation, vertical shift, and surface mismatch of the scan bodies was contrasted
Gedrimiene et al., 2019 [20]	To compare the accuracy of implant-supported FPDs by employing CIT and DIT techniques in the clinical setting	The laboratory scanner D800 (3Shape) was used to scan the master models. 3D models from the CIT and DIT workflows were transferred into reverse engineering software and placed on a scan body with high-resolution 3D CAD models. The amount of distance between the center points, angulation, rotation, vertical shift, and surface mismatch of the scan bodies was compared to one another
Alsharbaty et al., 2019 [21]	To evaluate the accuracy of a DIT compared to CIT techniques in clinical situations	Three implant impression strategies were used: traditional pick-up, transfer, and DIT for each patient, and intraoral reference models were recorded. For the reference models and conventionally manufactured working casts, CMM was utilized to keep track of linear displacement observations, inter-implant distances, and angular shifts. The digital STL files were evaluated using CATIA 3D assessment software for the same variables as the CMM observations
Wismeijer et al., 2014 [22]	To evaluate the patient’s perception of the variance between an analog impression and an IOS technique in implant-supported FPDs, as well as the time discrepancy between both methods	CIT and DIT were used to make the final impressions for implant-supported FPDs. Patients were subsequently prompted to complete a questionnaire on their experiences with both procedures. The time spent performing these two processes was also documented

Scan Accuracy

According to Hashemi et al., the CIT and DIT workflows had comparable characteristics in terms of impression accuracy, framework adaption, and reconstruction aesthetics [15]. The RCT by Gintaute et al. showed that total DIT displayed a poorer performance than the hybrid analog-digital approach. Additionally, DIT with IOS Trios 3 and Dental System Lab-Software outperformed IOS Virtuo Vivo with DWOS Lab-Software concerning transfer accuracy of laboratory cross-mounting [17]. According to Schmidt et al., there was a propensity for partially edentulous patients to have greater precision in DIT results when contrasted with completely edentulous patients in the aggregated data of edentulous scenarios [18].

The inconsistency of the scanned surface of the scan bodies of the DIT, on the other hand, was found to be double that of the CIT by Rutkunas et al. Particularly angular and vertical shift and positional discrepancies of the scan bodies in both approaches had substantial clinical significance [19]. Gedrimiene et al. indicated that the linear differences between DIT and CIT were of minimal therapeutic importance in the implant-supported FPDs. The surface mismatch, however, was greater for the DIT than the CIT [20].

Alsharbaty et al. recorded measurements of linear and angular displacement in three planes using a coordinate measuring device to assess the accuracy of a DIT employing Trios 3 Shape IOS, pick-up and transfer procedures, and between the CIT themselves. The DIT showed that there were angulation errors of 5° to 8.5°, inter-implant distance errors of 160 to 270 μm, and linear displacement errors of 270 to 450 μm. Thus, it was determined that the DIT was too inaccurate to be utilized to generate implant impressions for partially edentulous subjects [21].

Time Efficiency

Four studies included in the review employing DIT and CIT showed the entire fabrication time of implant-supported FPDs. The fabrication time employing the DIT compared to the CIT was considerably reduced, according to Hashemi et al. and Joda et al. [15,16]. According to Hashemi et al., the average clinical duration was not substantially different between the DIT (43.3 minutes) and CIT (41.4 minutes) [15]. However, the average laboratory time was considerably lesser in the DIT (134.9 minutes) than in the CIT (457.2 minutes). The average restoration fabrication time (178.2 minutes) was much lower in the DIT workflow than in the CIT workflow (498.9 minutes). According to Joda et al., the average impression-making time for the digital workflow group 1 was 8.6 minutes, and it was 17.3 minutes and 12.9 minutes for digital workflow group 2 and mixed analog-digital workflow, respectively [16]. The reported laboratory work times were 84, 170, and 155 minutes, while the reported time frames for prosthesis delivery were 4.9, 5.8, and 4.7 minutes, for complete digital workflow groups 1, 2, and the mixed analog-digital group, respectively [16].

On the contrary, two further studies revealed that the total amount of time required for CIT was less than the DIT workflow [17,22]. According to Gintaute et al., regardless of the workflow employed, it must be taken into account that for clinical routine practices chairside changes might be required for every second patient [17]. Wismeijer et al. used a questionnaire with a 10-point scale to compare how patients perceived both treatment methods holistically [22]. Despite patients expressing a strong general preference for the IOS, the total time investment for processing and delivery of the prosthesis was viewed more adversely for IOS in comparison with the CIT.

Quality Assessment of the Included Studies

Table 3 shows a summary of the Cochrane evaluation of the RoB for the two included RCTs [16,17].

**Table 3:** Risk of bias (RoB) of the reviewed randomized controlled trials. - = high RoB; + = low RoB; ? = unclear RoB

Author, year	Random sequence generation (selection bias)	Allocation concealment (selection bias)	Blinding of participants and personnel (performance bias)	Blinding of outcome assessment (detection bias)	Incomplete outcome data (attrition bias)	Selective reporting (reporting bias)	Other bias	Overall risk of bias
Joda et al., 2021 [16]	+	?	+	-	+	+	+	High
Gintaute et al., 2021 [17]	+	+	+	-	+	+	+	Moderate

One study was determined to have a high RoB as it showed a high risk for detection bias and an unclear RoB for allocation concealment [16]. Another RCT was disclosed to have high RoB for detection bias and was assigned to have moderate-quality evidence [17]. Table 4 presents a summary of the quality of evidence for six included prospective studies which were rated with eight stars, which signifies high-quality evidence. A direct comparison between the reviewed studies was not feasible as the selected studies were heterogeneous with varied study designs and outcome measures, and hence, a meta-analysis was not conducted.

**Table 4:** Risk of bias of the prospective clinical studies reviewed. *: present.

Author, year	Representativeness of the exposed cohort	Selection of the non-exposed cohort	Selection of the non-exposed cohort	Outcome of interest not present at the beginning of the study	Main factor for comparability	Other additional factors for comparability	Assessment of outcome	Follow-up long enough for outcome to occur	Adequacy of follow-up of cohort	Total score	Methodological quality
Hashemi et al., 2022 [15]	*	*	*	*	*	-	*	*	*	8	Good
Schmidt et al., 2021 [18]	*	*	*	*	*	-	*	*	*	8	Good
Rutkunas et al., 2020 [19]	*	*	*	*	*	-	*	*	*	8	Good
Gedrimiene et al., 2019 [20]	*	*	*	*	*	-	*	*	*	8	Good
Alsharbaty et al., 2019 [21]	*	*	*	*	*	-	*	*	*	8	Good
Wismeijer et al., 2014 [22]	*	*	*	*	*	-	*	*	*	8	Good

Discussion

The literature on the most recent data on scan accuracy and turnaround time for comparing IOS to CIT was analyzed in this systematic review. Clinical expertise did not have any major influence on the effectiveness of IOS, but novice clinicians may find CIT to be more challenging and time-consuming, which may have an impact on the final results. Another benefit of DIT utilizing an IOS is that it works well for patients who have profound vomiting reflexes and allows for selective overwriting of areas when the impression is unclear [23]. The overall fabrication time decreased when reconstructing and polymerizing procedures were taken into account [24]. There is very limited clinical evidence to advocate for employing IOS on implant-supported FPDs rather than CIT [25]. The therapeutic application of DIT in implant-supported FPDs exhibiting clinically tolerable differences was validated by this review.

Precision refers to the similarity of the findings gathered from various measurements, whereas accuracy relates to trueness, expressing the degree to which a measurement corresponds to the actual value [25,26]. Several studies have used study-specific reference models to examine the accuracy of various IOS systems. However, as DIT evolves, it turns out to be more challenging to generate a sufficient overview of scanning quality and to compare related research without a specific standardized baseline. Comparing various IOS technologies is further challenging due to the absence of standardized calibrations for the common laboratory and clinical processes used in the DIT workflow [27].

There was variation in time efficiency among the several studies. There was a definite trend for IOS to have less working time compared to CIT in two [15,16] of the four [15-17,22] studies evaluated. Six studies (75% of the reviewed studies) addressed the precision of the FPDs [15,17-21]. This was consistent with a previous review, which found that patient-oriented metrics, coupled with standard clinical criteria for the evaluation of FPDs, such as assessment of marginal integrity and occlusion, have emerged as essential for precision [8]. According to a recent assessment, IOS is associated with a shorter average retake time but a greater average retake frequency. This is because when employing DIT, professionals were more inclined to maximize the means of rescanning a missing area. In contrast, retaking a CIT would necessitate revisiting the entire process [2]. Furthermore, there were no standardized guidelines for measuring procedural working time [28].

The dearth of a standardized protocol for gauging the precision of intraoral impression techniques may be the primary impediment to accomplishing in vivo experiments. To obtain a reference model, the intraoral orientation of dental implants ought to be accurately captured and replicated in a model by employing any impression techniques. However, the approach for obtaining a reference model has been associated innately with inaccuracy, which generates a bias in the evaluation of the impression procedure that is to be evaluated. The variability of CIT outcome measures of in vivo studies illustrates that even the methods and materials used, namely, the design of the impression trays, implant coping, impression material, and splinting, contribute to the accuracy level of the reference frameworks. In vitro investigations, on the other hand, comprise reference and test models that are calibrated with the same tools, such as coordinate measuring machines, microscopes, digital micrometers, and standardized images. The accuracy is calculated as the difference between the test and reference framework models. The investigators of in vivo studies used diverse methods of evaluation to acquire data from the in vivo handling of CIT and DIT. A majority of in vivo studies did not provide quantitative information for determining the level of accuracy of impression techniques [26].

Trios, iTero, Cerec, and True Definition are the most frequently utilized intraoral scanners described in the literature for measuring completely and partially edentulous models. Trios was shown to have the best trueness score for the partially edentulous model, followed by True Definition, iTero, and Cerec [24]. Various factors, notably, the type of IOS system used, user expertise and comfort threshold, and the beginning and ending locations used for evaluating procedural working time, might have a detrimental effect on the effectiveness of the fabrication and delivery time of the prosthesis [2]. It should be highlighted that the information obtained from IOS is not intended to be deployed immediately in clinical practice but needs to be transformed into a digital form utilizing model builder software. This might mitigate acquisition mistakes during the matching phase of the CAD information of the scan body against the STL database of the scanner, contributing to a lower margin of error. Aside from data collection, data interpretation may also exert a significant impact on the degree of accuracy [10].

In the majority of the reviewed studies, DIT was captured utilizing a Trios 3 IOS. The juxtaposition of discrepancies between the two methodologies indicates that DIT was more accurate than CIT in only a few of the clinical studies reviewed [15,17,18]. In several other included studies, CIT was observed to be more accurate for partially edentulous jaws. The extent and region (anterior/posterior) of the edentulous area are considered pivotal criteria, indicating that a greater span length compromised the scan accuracy [29]. Other factors that can influence scanning outcomes include light reflection, operator motion, and restricted intraoral space. Furthermore, the computational integrity of the files generated through DIT, which is impacted by the accuracy and resolution of the scanner also plays an essential role. The precision of these scanners is critical as obtaining an exact impression is the initial step toward obtaining a good fit for the final prosthesis. A good scanner should be able to deliver excellent fidelity, precision, and resolution because these factors have a direct impact on the portrayal of the necessary information [30].

Although DIT will broaden its scope of applications to provide superior results, CIT will continue to play an essential role in prosthetic rehabilitation, and clinicians must evaluate each circumstance of the working environment [7,31]. Care should be taken when using IOS for conclusive impressions in instances of more than four-unit FPDs with long edentulous spans or movable soft tissues, particularly in mandibular arches [31]. IOS scanning accuracy is inextricably linked to both the software and hardware versions. Digital systems should be updated regularly to ensure a greater rate of transfer accuracy [32]. There is evidence that the scanning methodology affects the accuracy and precision of DIT. Specific clinical recommendations can barely be inferred from the data available. Further research with an emphasis on the in vivo application of CIT and DIT with methodological approaches that accurately evaluate impression accuracy is required in the future. Clinical investigations and RCTs are recommended to increase the degree of validation of the impression technique [33].

## Conclusions

The usage of IOS in clinical practice is highly demanding owing to its time efficiency and accuracy level in the fabrication of implant-supported FDPs with a more favorable patient perception. The findings of this review indicate the prospective applicability of future IOS systems. This, in turn, warrants continued development, and DIT could eventually replace CIT. The IOS was reported to be outstanding in terms of patient preferences and total fabrication time efficiency. Additional in vivo studies are needed to establish the therapeutic usefulness and time efficiency of integrating IOS in more comprehensive settings.
